# MARITAL BIOGRAPHY AND ACCELERATED AGING: EVIDENCE FROM THE HEALTH AND RETIREMENT STUDY

**DOI:** 10.1093/geroni/igad104.0321

**Published:** 2023-12-21

**Authors:** Won Choi, Haena Lee, Eileen Crimmins

**Affiliations:** University of Chicago, Chicago, Illinois, United States; Sungkyunkwan University, Seoul, Republic of Korea; University of Southern California, Los Angeles, California, United States

## Abstract

It is well-established that marriage promotes health and longevity. While a large body of research has examined marital status differences in health, no studies have focused on how marital status is linked to biological age. Moreover, the association between marital biography – transitions in and out of marriage across the life course – and biological aging also remains to be explored. Biological age, which refers to the “physiological functioning relative to the average physiological state at a specific chronological age,” has been steadily gaining attention as a measure indicative of an individual’s health status. In this study, we advance the literature on marital status and health by examining how marital status and marital transitions are associated with biological aging among a representative sample of U.S. older adults. We use an expanded measure of biological age that incorporates 22 biological indicators, more than half of which were not used in previous studies, to better assess how marital experiences shape accelerated aging. Using data from the 2016 Health and Retirement Study, we find that the pace of aging of those who have experienced one disruption is not statistically different from those continuously married. However, for both currently and previously married individuals, the experience of multiple marital disruptions is associated with accelerated aging. Health behaviors account for the association between multiple marital disruptions and accelerated aging for previously married older adults but not for currently married older adults. Psychosocial resources did not mediate the influence of marital biography on accelerated aging.

